# Categorization of Marketed Artificial Tear Formulations Based on Their Ingredients: A Rational Approach for Their Use

**DOI:** 10.3390/jcm10061289

**Published:** 2021-03-21

**Authors:** Avani Kathuria, Kiumars Shamloo, Vishal Jhanji, Ajay Sharma

**Affiliations:** 1 Chapman University School of Pharmacy, Chapman University, Irvine, CA 92618, USA; kathu101@mail.chapman.edu (A.K.); shaml103@mail.chapman.edu (K.S.); 2 Department of Ophthalmology, School of Medicine, University of Pittsburgh School of Medicine, Pittsburgh, PA 15213, USA; jhanjiv@upmc.edu

**Keywords:** artificial tears, dry eye, demulcents, ophthalmic preservatives, osmoprotectants, humectants

## Abstract

Dry eye disease is a common ocular condition affecting millions of people worldwide. Artificial tears are the first line therapy for the management of dry eye disease. Artificial tear formulations contain a variety of active ingredients, biologically active excipients, and preservatives. Many of these formulations are also available as preservative-free. This study was conducted to inspect artificial tear formulations currently marketed in the United States for their active ingredients, biologically relevant excipients, and preservatives. The marketed artificial tears were examined at various US retail pharmacy chains and using the manufacturers’ website to compile information about active ingredients, inactive ingredients, and preservatives. The currently marketed artificial tears can be grouped into four categories based on their active ingredients. The artificial tears also contain biologically active chemicals listed as inactive ingredients, which have osmoprotectant, humectant, and tear film lipid layer or mucous layer mimicking properties. Most artificial tears contain vanishing type preservatives such as purite or sodium perborate and safer quaternary compound polyquaternium-1. The majority of these artificial tear formulations are also available as preservative-free single dose unit. The study provides a formulary of artificial tears based on active ingredients, biologically active excipients, and the preservative-free option. The formulary should assist healthcare providers in making a stepwise and rational selection of appropriate artificial tears for patients suffering from dry eye disease.

## 1. Introduction

Dry eye disease is a common ocular condition affecting millions of people all over the world. According to National Health and Wellness Survey, an estimated 16.4 million people in the United States suffer from dry eye disease [1]. As per the current Tear Film & Ocular Surface Society (TFOS) definition, dry eye is a multifactorial disease characterized by loss of tear film homeostasis, tear hyperosmolarity, ocular surface inflammation, and neurosensory abnormalities [2]. The condition can cause a wide array of symptoms such as redness, stringy mucous, burning, and itchy sensation. Left untreated, the disease can result in visual disturbance and tear film instability with potential damage to the ocular surface [3]. Since the disease can significantly impact professional, social, and leisure activities, it leads the patient either to self-medicate with over-the-counter products or to seek medical help. Although the symptoms may improve or worsen over the natural course of the disease, dry eye disease is considered a lifelong condition [4]. Pharmacological and non-pharmacological interventions significantly improve the symptoms, however long-term adherence to these approaches is necessary for the effective management of dry eye disease. Detailed patient history, clinical examination, and diagnostic tests can play a very important role in effective management of this multifactorial disease [4]. A thorough clinical workup can also identify any underlying conditions or systemic diseases contributing to dry eye, since treating these underlying conditions is imperative for the effective management of dry eye disease [4].

Tear film is composed of a muco-aqueous layer underlying a lipid layer. It contains oxygen, metabolites, antimicrobials, and proteins [5]. The importance of tear film in keeping the ocular surface healthy has long been recognized, since it serves to provide nutrients, moisture, smooth the optical surface as well as helps to remove microbes. Tear replacement remains one of the mainstay approaches for the treatment of dry eye [4,6]. Although artificial tears are not meant to recapitulate the functions of natural tears, they do provide remarkable symptomatic and palliative relief in dry eye [4,6]. In 2018, artificial tears accounted for $2.2 billion in global sales, and their market continues to grow [7]. Healthcare providers play a critical role in guiding patients to choose the most appropriate artificial tear product. However, recommending the right artificial tear product can be difficult for many reasons. A vast number of artificial tear products are currently available in the United States. These artificial tears contain a wide variety of active ingredients and come in multidose vials with preservative or as preservative-free single dose units. Many of these artificial tears also contain excipients that are listed under inactive ingredients, but these excipients are known to contribute to their therapeutic benefits. Despite such a large variety of artificial tears, there is a paucity of information with up-to-date classification of marketed artificial tears based on their active and pharmacological relevant inactive ingredients, as well as their availability with or without preservatives. The aim of this study was to screen and categorize marketed artificial tear formulations based on their active ingredients, biologically relevant excipients, and preservatives. The information should help healthcare providers with the knowledge about which artificial tears they can initially choose and then switch to the ones with additional biologically active ingredients or another class with a different active ingredient.

## 2. Materials and Methods

The marketed artificial tears were surveyed at various US retail pharmacy chains and using the manufacturers’ website to compile the information about active ingredients, inactive ingredients, and preservatives. The artificial tear preparations were then tabulated based on the major active ingredient and the biologically active excipients listed under inactive ingredients. The type of preservative present in the formulations was noted, and it was also determined if these formulations were available as preservative-free single dose application units.

## 3. Results

### 3.1. Active Ingredients in Artificial Tears

Artificial tears contain one or more FDA-approved demulcents as active ingredient [8]. FDA defines demulcent as a primarily water-soluble polymer, which is applied topically to the eye to protect and lubricate mucous membrane surfaces and relieve dryness and irritation. The list of these demulcents and their concentration range was provided by the FDA in a 1988 monograph to help reduce the costs and expedite approval of artificial tears [8]. The FDA approved demulcents include (a) Cellulose derivatives: Carboxymethylcellulose (CMC), Hydroxypropyl methylcellulose (hypromellose), Hydroxyethylcellulose, Methylcellulose, (b) Dextran 70, (c) Gelatin, (d) Polyols: Glycerin, Polyethylene glycol (300, 400), Polysorbate 80, (e) polymers: Polyvinyl alcohol, Polyvinyl pyrrolidone (povidone).

Our screening data demonstrates that the currently marketed tears contain CMC, hypromellose, dextran, glycerin, polyethylene glycol 400, polysorbate 80, polyvinyl alcohol, and povidone as active ingredients (Table 1, Table 2, Table 3 and Table 4). However, there are no currently marketed artificial tears that contain hydroxyethyl cellulose, methylcellulose, gelatin, or polyethylene glycol 300. CMC is a polysaccharide with mucoadhesive properties and has been shown to bind to corneal epithelial cells. It enhances tear viscosity and increases the precorneal residence time of artificial tears at 1% concentration [9]. CMC is the primary active ingredient in Thera tears^®^ (Akorn Consumer Health, Ann Arbor, MI, USA) and Refresh^®^ brand (Allergan, Irvine, CA, USA) of artificial tears, with some formulations of the latter brand also containing glycerin and polysorbate 80 (Table 1). HPMC, better known as hypromellose, is also a polysaccharide polymer produced from cellulose. Hypromellose helps to moisten and lubricate the eye surface. Hypromellose along with dextran and glycerin are the demulcent and humectants in the GenTeal^®^ brand (Alcon, Fort Worth, TX, USA) of tears (Table 2). Further, our data analysis shows that propylene glycol, polyethylene glycol 400, and glycerin are three main polyol demulcents in currently marketed artificial tears (Table 3). Propylene glycol and glycerin also act as humectants to retain moisture at the ocular surface. The Systane^®^ brand (Alcon, Fort Worth, TX, USA) has two formulations: Systane Balance^®^ and Systane Complete^®^, with exact identical composition having propylene glycol as the active ingredient. However, these two formulations have significantly different physicochemical characteristics, with Systane Balance^®^ as microemulsion and Systane Complete^®^ as a nanodrop formulation [10]. Three other formulations from Systane^®^ are Original^®^, Ultra^®^, and Gel^®^, which contain propylene glycol plus polyethylene glycol as active ingredients but differ in their physicochemical properties— especially viscosity and biological properties—as well despite having a similar composition [10]. Soothe^®^ drops (Bausch + Lomb, Bridgewater, NJ, USA) also contain both glycerin and propylene glycol as its active ingredients (Table 3). Oasis^®^ brand drops (Oasis Medical, Inc., Glendora, CA, USA) and Blink^®^ tears (Johnson & Johnson Vision, Jacksonville, FL, USA) contain glycerin and polyethylene glycol alone, respectively as demulcent (Table 3).

Our screening shows that povidone is the primary demulcent in Soothe hydration^®^ and maximum hydration brand eye drops, while polyvinyl alcohol is the demulcent in Akorn^®^ artificial tears (Akorn, Lake Forest, IL, USA). Murine^®^ tears and FreshKote^®^ (Eyevance Pharmaceuticals, Fort Worth, TX, USA) use a combination of povidone and polyvinyl alcohol (Table 4).

### 3.2. Biologically Active Excipients in Artificial Tears

One downside of the FDA’s pre-approved monograph is that if a developer lists an active ingredient that is not on the FDA monograph, they need to provide clinical data proving the safety of its ophthalmic use in humans. Many pharmaceutical excipients and food additives have been shown to provide therapeutic relief in dry eye disease. Although these excipients and additives have ophthalmic therapeutic potential, the manufacturers list them under inactive ingredients in artificial tears to avoid trials. Because of their notable biological activity, health care providers need to be aware of these excipients and additives. Therefore, we carefully reviewed the biologically active pharmaceutical excipients and food additives present in these drops.

Our survey data shows that the currently marketed artificial tears use the following biologically active pharmaceutical excipients and food additives: (a) Osmoprotectants: trehalose, erythritol, levocarnitine, (b) Humectants: sodium hyaluronate, (c) Hydroxypropyl Guar gum, (d) Oils: flaxseed oil, castor oil, mineral oil.

Osmoprotectants are agents that protect cells against hyperosmolar stress-mediated injury. Since hyperosmolarity is one the characteristics of dry eye, osmoprotectants are emerging ingredients of artificial tears. Levocarnitine (L-carnitine) is present in prokaryotic and eukaryotic cells, including human corneal and conjunctival epithelia [11,12]. Erythritol is polyol used as a low-calorie sweetener. In vitro studies using cultured human corneal epithelial cells demonstrate that osmoprotectants are taken up by the dehydrated cells, then restore volume and prevent protein denaturation [13,14]. Trehalose is a naturally occurring disaccharide consisting of two alpha-glucose units joined by glucoside bond. Trehalose has also been shown to protect corneal epithelial cells from desiccation and high osmolarity [15,16,17]. Our data analysis demonstrated that erythritol, L-carnitine, and trehalose are present in some Refresh^®^ brand artificial tears, which contain CMC as the main demulcent (Table 1).

Humectants are hygroscopic agents that facilitate the retention of water. Hyaluronic acid is a high molecular weight naturally occurring glycosaminoglycan polysaccharide [18,19]. It is present in connective tissue, synovial fluid, and in the aqueous humor, and is vitreous [18,19]. Hyaluronic acid acts as a humectant and can bind water multiple times the amount of its weight. Sodium hyaluronate is a semisynthetic smaller molecular weight sodium salt derivative of hyaluronic acid. Like hyaluronic acid, sodium hyaluronate also acts as a humectant to provide additional hydration. Its mucoadhesive properties are proposed to increase the corneal residence time. Our data demonstrates that sodium hyaluronate is present in three artificial tear formulations with CMC, glycerin, and polyethylene glycol as demulcents (Table 1 and Table 3). It is worthwhile to note that in many artificial tears marketed in Europe and Asia (e.g., Hylo brand tears^®^, Vismed Multi^®^, Xailin HA^®^, Thealoz Duo^®^, BLUyal OSD^®^, Hyalistil Bio^®^, Artelac^®^ Splash™, Zolag^®^), sodium hyaluronate is listed as an active ingredient, because unlike the FDA, agencies in these countries do not have a list of demulcents for expediated approval.

Hydroxypropyl guar is a viscous protein polymer derived from beans. Upon instillation, hydroxypropyl guar forms a soft gel with increased viscosity at the tear pH. It mimics the properties of mucin layer by strengthening the attachment of the aqueous layer to the mucins and promotes the retention of other demulcents [20,21]. It has also been shown to stabilize the tear film. Hydroxypropyl guar is present in all Systane^®^ brand artificial tears (Table 3).

Flaxseed oil, castor oil, and mineral oil mimic the lipid layer of tear film, stabilize tear film, increase lipid layer thickness, and prevent evaporation [22,23,24]. Our survey demonstrates that among the currently marketed artificial tears, two Refresh brand formulations contain castor oil, flaxseed oil, and polysorbate 80 with CMC as a demulcent (Table 1). Two Systane^®^ brand formulations contain mineral oil and phospholipid dimyristoyl phosphatidylglycerol with propylene glycol as demulcent (Table 3). Polysorbate 80 and dimyristoyl phosphatidylglycerol in these formulations act as emulsifiers in these formulations, but also have additional biological advantage to help spread and stabilize the lipid layer upon instillation [10]. All these oils containing artificial tear formulations are emulsions.

### 3.3. Preservatives in Artificial Tears

Ophthalmic formulations are required to be sterile for the obvious reason of protecting the eye from infections. To maintain the sterility, artificial tears as a multidose ophthalmic formulation need to contain preservatives or alternatively need to be packaged as preservative-free single dose units. Based on our survey, the currently marketed artificial tears use purite, polyquaternium-1, sodium perborate, and benzalkonium chloride as preservatives with only one brand Blink^®^ Tears using sodium chlorite (Table 1, Table 2, Table 3 and Table 4). Many of these drops are also available in preservative-free single dose units. Refresh^®^ brand of tears contains purite as a preservative in their formulation but also offer most of their tears in a preservative-free form (Table 1). Sodium perborate is the preservative in Thera Tears^®^ and GenTeal^®^ brand tears (Table 1 and Table 2). Polyquaternium (Polyquad) is the preservative present in GenTeal^®^ and Systane^®^ brand tears. All the artificial tears with povidone and polyvinyl alcohol use benzalkonium chloride as a preservative except FreshKote^®^ which is available preservative-free (Table 4). Interestingly, Europe has many artificial tears available in multidose special ophthalmic dispensers. These multidose artificial tears are preservative-free, since the special dispensers are designed to prevent microbial contamination.

## 4. Discussion

Demulcents are the primary active ingredient for artificial tears. Our data analysis shows that there are primarily four categories of artificial tears to select from based on the demulcent present in them. Two brands contain CMC as demulcents, with Thera Tears^®^ containing the lowest concentration of CMC at 0.25%. These drops are a great option for patients with milder symptoms of dry eye who better tolerate lower concentrations of CMC. Thera Tears^®^ are available in multidose vial containing the vanishing preservative sodium perborate or as preservative-free single application. Thera Tears extra^®^ with 0.25% CMC plus trehalose as an additional ingredient are available only as sodium perborate preservative containing multidose vial. Sodium perborate, also known as Dequest, is a vanishing type of preservative. In solution, it is converted to hydrogen peroxide and causes the bactericidal effect by oxidative stress-mediated denaturation of bacterial proteins. Upon instillation into an eye, it is converted to oxygen and water by the enzyme catalase. Sodium perborate is less toxic than benzalkonium chloride but not completely innocuous [25,26]. Therefore, preservative-free Thera Tears^®^ should be preferred if a patient needs more than four applications per day.

Refresh^®^ brand of artificial tears have several formulations containing 0.5% CMC alone, with additional demulcents and biologically active excipients like osmoprotectants erythritol, l-carnitine, and trehalose. L-carnitine and erythritol have been shown to attenuate hyperosmolar stress-mediated increase in MAP kinases, pro-inflammatory cytokines, and matrix metalloproteases in corneal epithelial cells [27,28,29,30]. Clinical studies demonstrate that artificial tears containing L-carnitine and erythritol reduce tear osmolarity and provide symptomatic relief [31,32,33]. Studies in animal models of dry eye and clinical studies with trehalose containing eye drops demonstrate many beneficial effects, including increased tear production, tear stabilization, reduced corneal staining, and a decrease in corneal epithelial cell apoptosis [34,35,36,37]. Humectant sodium hyaluronate in Refresh^®^, Oasis^®^ and Blink^®^ tears can offer significant additional relief in dry eye in preventing or treating corneal epithelial damage since in vitro studies demonstrate that sodium hyaluronate promotes corneal epithelial cell migration and proliferation, thus facilitating healing [38,39,40]. Topical application of sodium hyaluronate in rabbits and mice has been shown to promote wound healing and relieve dry eye symptoms [41,42]. Castor oils and flaxseed oil in some Refresh^®^ Brand tears will offer the additional benefit of reduced tear evaporation in patients with evaporative dry eye. Purite, the preservative present in Refresh^®^ brand artificial tears, is stabilized oxychloro complex (SOC) containing 99.5% chlorite, 0.5% chlorate. In solution, its bactericidal action is mediated by the oxidizing action of chlorine dioxide free radical. It belongs to the vanishing class of artificial tears since upon instillation, it is converted to sodium chloride, oxygen, and water [43,44]. Animal studies demonstrate that purite is significantly less toxic to corneal epithelium compared to benzalkonium chloride but has some degree of detrimental effect compared to preservative-free artificial tears [43,44,45,46,47]. Given the wide variety of options, a patient can be started on Refresh^®^ tears with CMC alone either with or without preservatives depending upon the frequency of instillation required and if the dry eye relief is inadequate, Refresh^®^ tears with additional biologically active excipients can be tried.

The Systane^®^ brand of tears use propylene glycol and glycerin as demulcent and humectants to retain moisture at ocular surfaces along with dimyristol phosphatidyl glycerol, hydroxy propyl guar, and mineral oil listed under inactive ingredients purportedly mimicking the mucin and the lipid component of tear film and polyquaternium-1 as a preservative. In an in vitro model of 3D corneal epithelium, the dual polymer formulation of hydroxypropyl guar/sodium hyaluronate demonstrated enhanced the ocular surface hydration, surface retention, cell barrier function, and lubricity compared to single-polymer formulations [19,20]. Polyquaternium (Polyquad) is a commonly used preservative in Systane^®^ ophthalmic drops. This compound is a quaternary ammonium compound and has bactericidal activity due to the surfactant action just like benzalkonium chloride. However, this compound is a large size polymer and is safe for mammalian cells, apparently due to its reduced penetration owing to its larger size [43,48]. Several in vitro and animal studies have compared the ocular surface toxicity of polyquaternium and benzalkonium chloride. In vitro studies demonstrate that polyquaternium has low cytotoxicity and it is remarkably safer than benzalkonium chloride [49,50,51,52]. Further, it seems to be well tolerated upon topical instillation in animal studies and in patients taking polyquaternium preserved glaucoma drops [53,54,55,56].

Benzalkonium chloride is another common preservative used in ophthalmic drops. However, our data reveals that among the current brand name artificial tears, only Soothe^®^, Akron^®^, and Murine^®^ brand tears use benzalkonium chloride as a preservative. Benzalkonium chloride is an amphiphilic quaternary ammonium compound that kills bacteria by surfactant action on their plasma membrane. However, mammalian cells, including the corneal and conjunctival epithelial cells can also be impacted by the surfactant action of benzalkonium chloride. In vitro studies using corneal and conjunctival epithelial cells demonstrate that benzalkonium chloride decreases ocular surface epithelial cell viability and increases the release of pro-inflammatory mediators [57,58,59,60,61]. Topical application of benzalkonium chloride in rabbits and rodents has been demonstrated to cause tear film abnormalities, reduction in mucins and goblet cell density, corneal epithelial desquamation, and an influx of inflammatory cells [62,63,64,65,66]. Further studies in rodents revealed detrimental changes in corneal innervation after topical application of benzalkonium chloride [67,68]. Glaucoma patients using benzalkonium chloride-containing eye drops have been reported to suffer from reduced tear film and tear breakup time, as well as increased tear osmolarity [43,69]. As observed in the animal studies, prolonged use of benzalkonium chloride containing eye drops in glaucoma patients has also been shown to cause damage to the ocular surface epithelial cells and corneal nerves [43]. Since many of the observed toxic effects of benzalkonium chloride can exacerbate ocular surface damage and further worsen the inflammatory response, the current consensus is to avoid the use of benzalkonium chloride containing artificial tears in dry eye patients.

## 5. Conclusions

The goal of screening the marketed artificial tear formulations was to give health care providers a formulary of artificial tears classified based on their active ingredients and biologically active excipients. The options of preservative-free single dose units, if available, has also been included in our data tables. The compiled information should assist the healthcare providers to make a stepwise and rational selection of appropriate artificial tears for the patients based on the active ingredients and biologically active excipients.

Due to the multifactorial etiology of dry eye, it is challenging to make generalized recommendations about selecting appropriate artificial tears. However, based on recent studies and clinical experience, some broad suggestions are available to make a judicious selection [37,70,71,72,73,74,75]. Individualized artificial tear therapy needs to be based on patient’s history, ocular examination, and clinical testing. Patients having mild increase in tear osmolarity and normal tear break up time (TBUT) can be managed with artificial tears containing one or two demulcents such as CMC (Thera Tears^®^; Refresh Tears^®^), hypromellose plus dextran (GenTeal Mild^®^), or polyols (Soothe^®^, Systane Original^®^, Systane Ultra^®^). Thera tears are hypo-osmolar and have a slightly alkaline pH that may offer a more soothing effect for some patients. If monitoring reveals inadequate response, formulation with added excipients such as osmoprotectants and sodium hyaluronate can be tried (Thera Tears Extra^®,^ Refresh Optive^®^, Refresh Repair^®^, Oasis^®^, Blink Tears^®^). Awareness about artificial tears based on different demulcents as active ingredients can help one make a rational switch from one class of active ingredient to another class in the case of inadequate relief. Cases with a moderate increase in osmolarity and quick TBUT may denote significant evaporative dry eye. Dry eye due to meibomian gland dysfunction also has significant evaporative component due to deficiency in the lipid layer. In such cases, artificial tears that contain lipid components including castor oil (Refresh Optive Advance^®^), flaxseed oil (Refresh Optive Omega 3^®^), mineral oil, and dimyristoyl phosphatidyl glycerol (Systane Balance^®^, Systane Complete^®^) that either replenish or stabilize the lipid layer should be considered. Formulations with unique bioactive excipients, such as dimyristoyl phosphatidyl glycerol and hydroxypropyl guar, may work better for some patients (Systane Original^®^, Systane Ultra^®^, Systane Balance^®^, Systane Complete^®^). The awareness about the availability of preservative-free formulations in each category is also critical. The option should be discussed with the patient whether they can afford preservative-free artificial tears and are willing to try them. Preservative-free formulations are recommended for patients requiring more than four to six applications per day.

Cases with moderate to high increase in osmolarity and significant ocular surface staining invariably require prescription based anti-inflammatory drops. The importance of lifestyle and dietary modification should be emphasized during counseling and is helpful for all types of dry eye patients. Warm compresses and lid hygiene are also critical for meibomian gland dysfunction. Use of gels and nighttime ointments is also an option for moderate cases. Ointments are exclusively for nighttime use but some gel formulations (Refresh Liquigel^®^, Refresh Celluvisc^®^, Refresh Optive Gel^®^, Systane Gel Drops^®^, Blink Gel Tears^®^) can be used during both the day and nighttime. Patients need to be counselled on transient blurring of vision with the daytime use of gels.

Several clinical studies have tested the efficacy of artificial tears in patients suffering from dry eye. These trials have been extensively reviewed [37,70,71,72,73,74,75]. Although no conclusive recommendations demonstrate the efficacy of one class of artificial tears is superior over another class due to variability in the study design, tested clinical end points, patients’ heterogeneity, and sponsor bias [70,71], nonetheless, these trials consistently demonstrate that artificial tears show therapeutic benefits and are useful armaments in the management of dry eye.

## Figures and Tables

**Table 1:** Artificial tears containing Carboxymethylcellulose (CMC) as a demulcent.

Active Ingredient(s)	Inactive Ingredients with Biological Activity	Brand	
		With Preservative	Preservative Free
CMC 0.25%		Thera Tears^®^ (Dequest)	Thera Tears^®^
CMC 0.25%	Trehalose	Thera Tears Extra^®^ (Dequest)	
CMC 0.5%		Refresh Tears^®^ (Purite)	Refresh Plus^®^
CMC 0.5% Glycerin 0.9%	Erythritol L-Carnitine	Refresh Optive^®^ (Purite)	Refresh Optive^®^
CMC 0.5% Glycerin 0.9%	Erythritol Sodium hyaluronate	Refresh Repair^®^ (Purite)	
CMC 0.5% Glycerin1% Polysorbate80 0.5%	Erythritol L-Carnitine Castor oil	Refresh Optive advanced^®^ (Purite)	Refresh Optive advanced^®^
CMC 0.5% Glycerin 1% Polysorbate80 0.5%	Erythritol L-Carnitine Trehalose Castor oil Flaxseed oil		Refresh Optive Mega-3^®^
CMC 1%		Refresh Liquigel^®^ (Purite),	Refresh Celluvisc^®^, Thera tears Liquigel^®^
CMC 1% Glycerin 0.9%	Erythritol L-Carnitine	Refresh Optive Gel^®^ (Purite)	

**Table 2:** Artificial tears containing hypromellose and dextran as demulcents.

Active Ingredient(s)	Brand
Hypromellose 0.3% Dextran 0.1%	GenTeal Mild^®^ (Polyquaternium-1)
Hypromellose 0.3% Dextran 0.1% Glycerin 0.2%	GenTeal Moderate^®^ (Polyquaternium-1) Also available as preservative free
Hypromellose 0.3% + Carbopol 980 Hypromellose 0.3%	GenTeal Severe^®^, Systane lubricant eye gel^®^ (Sodium perborate)

**Table 3:** Artificial tears containing propylene glycol, polyethylene glycol, and glycerin as demulcents.

Active Ingredient(s)	Inactive Ingredients with Biological Activity	Brand	
		With Preservative	Preservative Free
Propylene Glycol 0.6%	Dimyristoyl phosphatidyl glycerol Hydroxypropyl guar Mineral oil	Systane Balance^®^ Systane Complete^®^ (nanodrops) (Polyquaternium-1)	
Propylene Glycol 0.3% Polyethylene Glycol 0.4%	Hydroxypropyl guar Sodium hyaluronate (only in Hydration^®)^	Systane Original^®^ Systane Ultra^®^ Systane Gel Drops^®^ (Polyquaternium-1)	Systane Original^®^ Systane Ultra^®^ Systane Hydration^®^
Glycerin 0.22–0.25%	Sodium hyaluronate		Oasis tears^®^ Oasis tears plus^®^
Polyethylene Glycol 0.25%	Sodium hyaluronate	Blink Tears Blink Gel Tears^®^ (Sodium Chlorite)	Blink Tears^®^
Glycerin 0.6% Propylene Glycol 0.6%			Soothe^®^

**Table 4:** Artificial tears containing povidone and polyvinyl alcohol as demulcents.

Active Ingredient(s)	Brand	
	With Preservative	Preservative Free
Povidone 1.25%	Soothe Hydration^®^ (EDTA)	
Povidone 2%	Soothe maximum hydration^®^ (Benzalkonium Chloride)	
Polyvinyl alcohol 1.4%	Akorn^®^ artificial tears (Benzalkonium Chloride)	
Povidone 0.6% Polyvinyl alcohol 0.5%	Murine^®^ tears (Benzalkonium Chloride)	
Povidone 2% Polyvinyl alcohol 2.7%		FreshKote^®^
